# Preparation and Properties of Porous Concrete Based on Geopolymer of Red Mud and Yellow River Sediment

**DOI:** 10.3390/ma17040923

**Published:** 2024-02-17

**Authors:** Yajun Lv, Yiming Chen, Wei Dai, He Yang, Linhua Jiang, Keliang Li, Weizhun Jin

**Affiliations:** 1 School of Architecture, North China University of Water Resources and Electric Power, Zhengzhou 450046, China; lvyajun@ncwu.edu.cn (Y.L.); chenyiming0528@163.com (Y.C.); 2 Transportation Development Center of Henan Province, Zhengzhou 450016, China; hnmsajhcdw@163.com (W.D.); yanghe8813@163.com (H.Y.); 3 College of Civil and Transportation Engineering, Hohai University, Nanjing 210024, China; 4 School of Civil Engineering and Communication, North China University of Water Resources and Electric Power, Zhengzhou 450045, China; klli73@163.com

**Keywords:** red mud, Yellow River sediment, geopolymer, porous concrete, heavy metal adsorption

## Abstract

Red mud (RM) and Yellow River sediment (YRS) are challenging to handle as waste materials. In this study, RM with geopolymer and heavy metal adsorption characteristics was combined with YRS and ground granulated blast furnace slag (GGBS) to develop a porous geopolymer with high strength and high adsorption performance. A geopolymer cementitious material with high strength was prepared using high temperature water bath curing of 90 °C and different dosages of YRS, and a porous geopolymer concrete was further prepared. The compressive strength, fluidity and setting time of geopolymer cementitious materials were tested, and the compressive strength, porosity and permeability of porous geopolymer concrete were also tested. The environmental impact assessment of geopolymer cementitious materials was further conducted. The hydration products and microstructure of geopolymer gel materials were analyzed by XRD, SEM and FT-IR tests. The results show that the addition of YRS can effectively prolong the setting time of the geopolymer cementitious material, and the enhancement rate is as high as 150% compared with the geopolymer cementitious materials without the addition of YRS. An appropriate amount of YRS can improve the compressive strength of the geopolymer cementitious materials, and its early compressive strength can be further improved under the high temperature water bath curing of 90 °C, and the compressive strength at an age of 3 d can be up to 86.7 MPa. Meanwhile, the compressive strength of porous geopolymer concrete at an age of 28 d is up to 28.1 MPa. YRS can participate in geopolymer reactions, and high temperature water bath curing can promote the reaction degree. Curing method and YRS dosages have little effect on the porosity and permeability of the porous geopolymer concrete. The porous geopolymer has a good heavy metal adsorption effect, and the alkaline pH values can be gradually diluted to neutral.

## 1. Introduction

In recent years, the rapid advancement of urbanization has led to a sharp increase in building density and land surface coverage, triggering a transformation in the urban surface water circulation system and frequent occurrence of internal flooding [1]. Porous concrete, known for its excellent permeability and drainage capacity [2,3,4,5], is typically composed of cement mortar and aggregates and widely applied in areas such as parking lots, permeable road surfaces, and riverbank slopes [6,7,8,9,10]. With its unique porous structure, porous concrete plays a crucial role in mitigating urban flood risks and alleviating the urban heat island effect [11,12]. However, the cement in porous concrete is not only the world’s most important building material, but also a major source of carbon dioxide emissions. Therefore, the extensive use of concrete will lead to the deterioration of the ecological environment [13,14,15,16,17]. To address the environmental pollution associated with traditional concrete, an increasing number of researchers have turned their attention to the study of geopolymer-modified concrete. Geopolymer usually consists of the precursor materials of geopolymer containing Si, Al and Ca, such as blast furnace slag, metakaolin and fly ash, which can be combined with an alkali activator to prepare geopolymer gels with three-dimensional network structure. These geopolymer gels, with their excellent engineering performance, can replace traditional concrete and reduce carbon emissions during concrete production [18,19,20,21,22].

Red mud (RM) is an industrial by-product of alumina production. The amount of RM ranges from 1.5 to 2.5 tons per ton of aluminum produced, the amount of RM ranges from 1.5 to 2.5 tons per ton of aluminum produced, and the annual global output of RM is about 120 million tons per year, [23], its toxicity and radioactivity pose a threat to the living environment [24,25]. Henan province of China is a major region of aluminum production, with an annual output of about 15 million tons of RM. It is mainly disposed of by RM storage, with a stockpile of about 240 million tons, accounting for more than a quarter of the country. The Yellow River, flowing through Henan province, is the largest sediment-laden river around the world, which contributes to 6% of the global sediment transport [26]. The accumulation of sediment will raise the river bed and increase the risk of flooding. At present, how to effectively dispose the Yellow River sediment (YRS) is a great challenge. Therefore, the utilization of RM and YRS instead of cement in the preparation of porous geopolymer concrete holds significant ecological significance for reducing the accumulation of RM and YRS, and improving the ecological environment.

As the main components of RM are composed of the residue of bauxite after extracting alumina and the auxiliary materials (such as alkali and lime) added in the smelting process, the main chemical compositions are SiO_2_, CaO, Al_2_O_3_, etc., which have high activity and specific surface area, and thus can be applied to the preparation of geopolymer cementitious materials. Several studies have shown that RM can be used as a precursor material for geo-polymerization. Zhang et al. [27] demonstrated that RM can combine with fly ash to generate geopolymers with strength ranging from 7 to 13 MPa. Chen et al. [28] utilized RM, ultrafine fly ash, and recycled concrete to prepare cement-free ternary geopolymer composite materials with a strength of up to 46 MPa. Singh et al. [29] activated Bayer process RM and combined it with fly ash to produce a geopolymer with a 7 d strength of 40 MPa. An et al. [30] combined RM with blast furnace slag and calcium carbide slag to create geopolymer, and the strength could reach 20.3 MPa at 28 d when the RM content is 30%. Although RM was used in the preparation of geopolymer in the above studies, the strength achieved was relatively low. Calcination-produced RM exhibits high reactivity and a high calcium content, making it suitable as a base material for geopolymers, especially when combined with ground granulated blast furnace slag (GGBS), which results in a higher strength. GGBS is a by-product of pig iron smelting, and it shows good performance when combined with other geopolymer raw materials [31]. Zhang et al. [32] used RM and blast furnace slag to prepare ultra-high-performance geopolymer with a strength of 118 MPa. However, the 12-minute setting time renders it impractical for the construction of porous concrete in real engineering projects. Therefore, extending the setting time of high-performance geopolymer concrete has become a challenge. In view of this problem, a variety of retarders for silicate materials in the existing literature cannot prolong the setting time of geopolymer, which has become a major obstacle to the development of porous geopolymer concrete.

This study proposes the substitution of YRS for RM and slag in the preparation of ternary geopolymer cementitious materials, followed by the further production of porous geopolymer concrete. High-temperature water bath curing and standard curing will be applied to the geopolymer cementitious materials and porous geopolymer concrete. The compressive strength, flowability, and setting time tests will be conducted on geopolymer cementitious materials. Compressive strength, porosity, and permeability tests will be conducted on the porous geopolymer concrete. The heavy metal adsorption performance and environmental pH values of geopolymer cementitious materials were tested. This research aims to explore the feasibility of using RM and YRS as geopolymer cementitious materials and assess the feasibility of the high temperature water bath curing method.

## 2. Materials and Methods

### 2.1. Materials

**Figure 1:**
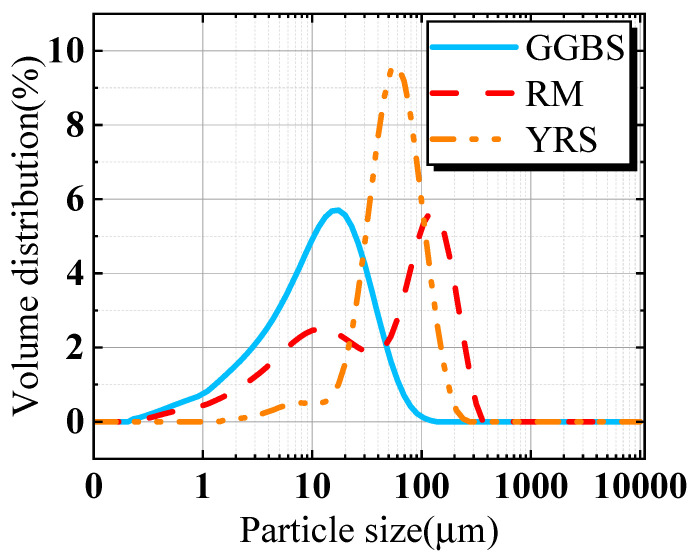
The volume distribution curves of GGBS, RM and YRS.

Sintered RM is supplied by the Zhongzhou Branch of Aluminum Corporation of China (Jiaozuo, China). After drying, it is subjected to 30 min of ball milling and sieving (0.16 mm). YRS is collected from the coastal area along the downstream Puyang section of the Yellow River. After drying, it is sieved through a mesh (0.075 mm). Ground granulated blast furnace slag (GGBS) is provided by Longze Water Purification Materials Co., Ltd. in Zhengzhou, China. Figure 1 presents the particle size distribution test results for RM, YRS and GGBS. A sodium silicate solution, combined with sodium hydroxide and adjusted to a mol of 2.1 (SiO_2_: Na_2_O = 2.1), is selected as the alkali activator and provided by Henan Borun Casting Materials Co., Ltd., Zhengzhou, China. Gravel with an average particle size of 5 mm is used as the aggregate, washed to remove the surface dust and dried. Three of each test block were prepared to reduce the test error and to calculate the standard deviation.

**Figure 2:**
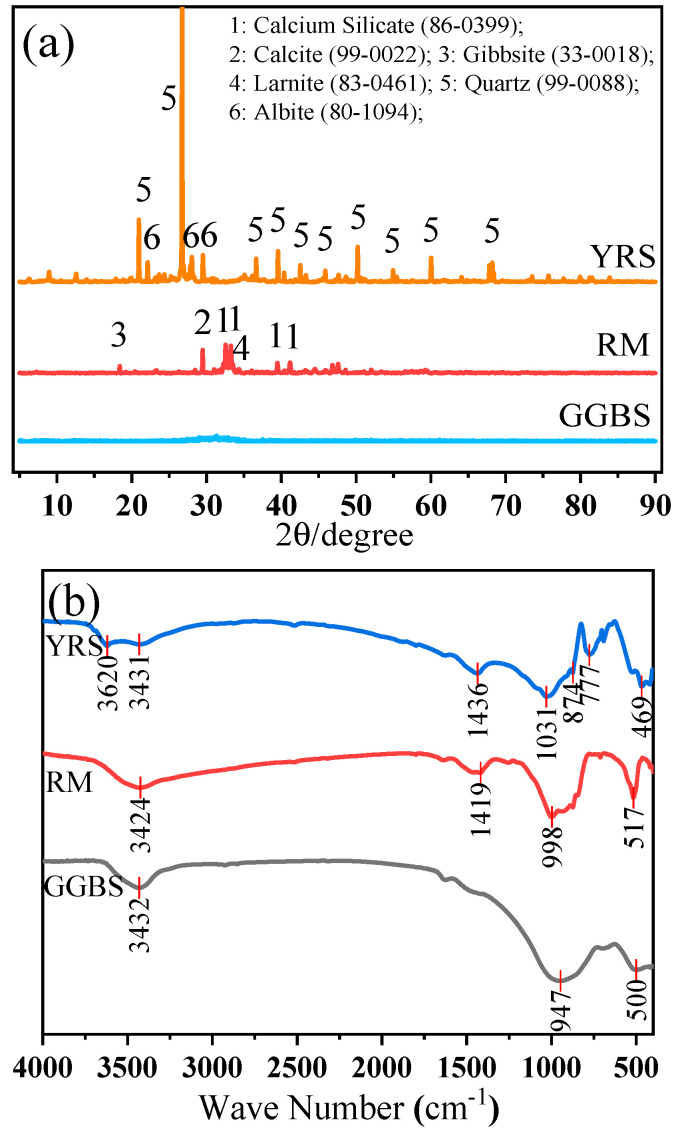
XRD patterns (**a**) and FT-IR spectrums (**b**) of RM, GGBS and YRS.

Chemical compositions of RM, GGBS, and YRS were conducted using X-ray fluorescence spectrometer (XRF, Panalytical Axios, PANalytical B.V., Almelo, The Netherlands), X-ray diffraction (XRD, Rigaku Ultima IV X, Rigaku, Tokyo, Japan), and Fourier-transform infrared spectroscopy (FT-IR, Thermo Scientific Nicolet iS20, Waltham, MA, USA). The test results are presented in Table 1 and Figure 2. From Table 1, it is observed that the RM and GGBS have higher CaO compared with YRS, indicating higher reactivity of the RM and GGBS than that of YRS. As shown in Figure 2a, the mineral components in RM mainly include the calcium silicate, calcite, gibbsite and diaspore, while YRS mainly contains quartz and sodium feldspar. GGBS exhibits a broad peak distribution between 20° and 36°, indicating mostly amorphous phases [33]. As can be seen from Figure 2b, the absorption peaks at 469~1031 cm^−1^ are Si-O-Si stretching vibration, 1419~1436 cm^−1^ are C=O stretching vibration, and 3424~3620 cm^−1^ are O-H stretching vibration [28].

### 2.2. Mix Design

The mass ratio of GGBS to RM is fixed at 3:7. YRS is used to replace GGBS and RM in equal masses to prepare the geopolymer cementitious materials and porous geopolymer concrete with added aggregates. Two different curing methods are employed: standard curing and a combination of high temperature water bath curing of 90 °C for 3 d and followed by standard curing. The curing temperature of 90 °C is based on the existing research of geopolymers [34]. The mix proportions are detailed in Table 2. Na_2_SiO_3_ with a mol of 2.3 is chosen as the alkali activator, and NaOH is used to adjust the mol to 2.1. To maintain the consistent fluidity of the slurry, the water-to-binder ratios are set between 0.36 and 0.4. Since the fluidity significantly influences the performance of porous geopolymer concrete, the water-to-binder ratios are selected to ensure the constant slurry fluidity. Considering that the optimal permeability of porous concrete is achieved when the porosity ranges between 10% and 30%, and the strength requirements are satisfied within this range, the aggregate quantity is determined based on a designed permeability rate of 15%, following the fixed amount of binder materials [35].

### 2.3. Sample Preparation

The method of preparing porous geopolymer concrete involves encapsulating aggregates with geopolymer cementitious materials. The sample preparation steps are as follows: (a) Na_2_SiO_3_ and NaOH were mixed with water and allowed to dissolve completely to form a base activator solution; (b) GGBS, RM and YRS were mixed and stirred evenly, then the alkali activator solution was added and stirred for 2 min before pouring into the mold to form the geopolymer cementitious materials; (c) the freshly prepared geopolymer cementitious material slurry was mixed with aggregates, uniform stirring ensured complete encapsulation of the aggregates with the geopolymer cementitious material slurry. Subsequently, the encapsulated aggregates were placed into molds in three installments. After each installment, the porous geopolymer concrete was prepared by manual vibration of iron rods 25 times each time and flattening.

**Figure 3:**
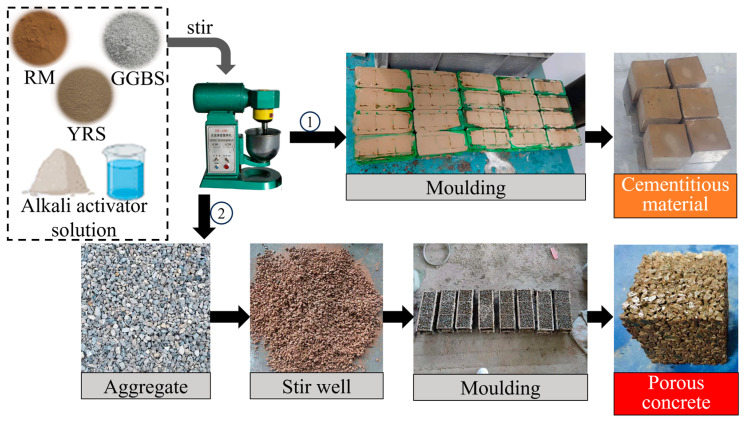
Preparation process of geopolymer cementitious materials and porous geopolymer concrete.

The samples, along with the molds, were covered together with plastic film and placed in a curing room with a humidity higher than 95% and a temperature of (20 ± 2) °C. After curing for 24 h, the samples were removed, and the molds were dismantled. The samples of Group H were placed in an oven of 90 °C for water bath curing for 3 d before transferring to the standard curing room. The samples of Group S were directly placed in the standard curing room. Both groups were then cured until needed before being taken out for testing. The casting dimensions for the geopolymer cementitious materials are 40 mm × 40 mm × 40 mm, and the casting dimensions for porous geopolymer concrete are 100 mm × 100 mm × 100 mm. The preparation process for the casting dimensions and porous geopolymer concrete is illustrated in Figure 3.

### 2.4. Test Methods

#### 2.4.1. Flowability and Setting Time Tests

The flowability and setting time of the geopolymer cementitious material slurry is measured according to the test methods outlined in GB/T 8077-2012 “Methods for testing uniformity of concrete admixture” and GB/T 1346-2011 “Test methods for water requirement of normal consistency, setting time and soundness of Portland cement” [36,37].

#### 2.4.2. Compressive Strength Test

The compressive strength of the geopolymer cementitious materials and porous geopolymer concrete is tested using an electro-hydraulic servo pressure testing machine in accordance with the standards GB/T 17671-1999 “Method of testing cements-Determination of strength” and GB/T 50081-2019 “Standard for test methods of concrete physical and mechanical properties” [38,39].

#### 2.4.3. Porosity and Water Permeability Tests

In designs of mixed proportions, the target porosity of pervious concrete is determined using the volumetric method. When calculating the quantities of various materials, the first step is to establish the target porosity, coarse aggregate packing density, and water-cement ratio. The calculation formula is as follows in Equation (1):

(1) $$\frac{m_{G}}{\rho_{G}}+\frac{m_{C}}{\rho_{C}}+\frac{m_{W}}{\rho_{W}}+P=1$$

where, $m_{G}$, $m_{C}$, and $m_{W}$ denote the quantities of aggregates, cementitious materials, and water per cubic meter of concrete, respectively, in kilograms, kg; $\rho_{G}$, $\rho_{C}$, and $\rho_{W}$ represent the densities of aggregates, cementitious materials, and water, respectively, in kilograms per cubic meter, kg/m^3^ and P is the target porosity, expressed as a percentage, %. The quantity of permeable concrete aggregates per cubic meter is determined by Equation (2):

(2) $$W_{G}=a\cdot \rho_{G}^{e}$$

where, $W_{G}$ represents the mass of aggregates in permeable concrete, measured in kilograms, kg; $\rho_{G}^{e}$ stands for the mass of aggregates under compacted conditions, measured in kilograms per cubic meter, kg and a is the correction factor for the quantity of aggregates, with a value of 0.98 for gravel.

The method for testing the actual density of porous geopolymer concrete is as follows: (a) retrieve the cured specimens after 28 d from the curing chamber and weigh them, submerge the specimens in water for 24 h, weigh them again while submerged, and finally, remove the specimens, dry them at 80 °C for 24 h, and weigh them once more; (b) divide the mass of the specimen by its theoretical volume to obtain the density of the specimen. The calculations for the total porosity and effective porosity of porous geopolymer concrete are given by Equations (3) and (4) [40]:

(3) $$K_{a}=\left(1-\left(\frac{M_{2}-M_{1}}{\rho_{w}V}\right)\right)\times 100\%$$

(4) $$K=\left(1-\left(\frac{M_{3}-M_{1}}{\rho_{w}V}\right)\right)\times 100\%$$

where, $K_{a}$ represents the total porosity, expressed as a percentage, %; K represents the effective porosity, expressed as a percentage, %; V is the apparent volume of the sample, m^3^; $M_{1}$ is the mass of the sample weighed in water after soaking for 24 h, measured in kilograms, kg; $M_{2}$ is the constant mass of the specimen after drying at 80 °C for 1 d, measured in kilograms, kg; $M_{3}$ is the mass of the specimen after 24 h of air exposure under standard curing conditions, measured in kilograms, kg and $\rho_{w}$ is the density of water, kg/m^3^.

Regarding the permeability calculation, the variable head method is employed to determine the permeability coefficient using a self-made simple permeability device. The device consists of a transparent plastic square tube with internal dimensions of 100 mm × 100 mm × 400 mm. The opening has a square shape measuring 100 mm × 100 mm, with markings on its front face. During testing, the tube is placed above the porous geopolymer concrete specimen. The side of the specimen is sealed with waterproof tape and connected to the tube. At the beginning of the experiment, first, the water was continuously added to the square tube until the scale of 350 mm, then the water was stopped. Then, the liquid level was observed to drop to $V_{1}$ (300 mm) and the timing was started ($T_{1}$), and $T_{2}$ was when the water level dropped to $V_{2}$ (100 mm). The permeability coefficient is calculated as shown in Equation (5):

(5) $$P=\frac{V_{1}-V_{2}}{T_{2}-T_{1}}$$

In the equation, P represents the permeability coefficient, measured in millimeters per second, mm/s.

#### 2.4.4. X-ray Diffraction, Scanning Electron Microscope and Fourier-Transform Infrared Tests

To further investigate the microscopic properties of samples, a series of microscopic tests were conducted for analysis. Prior to testing, the specimens were crushed, immersed in anhydrous ethanol to halt the hydration reaction, and dried under 60 °C for 2 d before experimentation. X-ray diffraction (XRD) was employed to test the powder formed from the crushed geopolymer cementitious materials. The scanning angle range was selected as 5–90°, with a scanning rate of 2°/min, a wavelength of 1.5418, and X-ray voltage and current set as 40 kV and 40 mA, respectively. Scanning electron microscopy (SEM, MIRA LMS, Tescan) was used to conduct microscopic morphology tests on the blocks formed from the crushed geopolymer cementitious material. The acceleration voltage range was 0.53 kV, and the low vacuum pressure was maintained between 1270 Pa. FT-IR was applied to analyze the chemical bonds in the sample powders, with a wavelength range from 400 to 4000 cm^−1^.

#### 2.4.5. Heavy Metal Adsorption and pH Tests

To investigate the adsorption impact of RM geopolymers on heavy metal ions, geopolymer cementitious materials were poured into a heavy metal solution, and immersed for 30 min for adsorption. Subsequently, the deionized water was used to rinse off the surface residues of unadsorbed firmly bound heavy metal elements. The surface was then subjected to Energy Dispersive X-ray Spectroscopy (EDS) scans. Metal solutions were prepared using CdCl_2_ (cadmium chloride), PbCl_2_ (lead chloride), CuCl_2_ (copper chloride), and deionized water. The concentration of each heavy metal ion (Cd^2+^, Pb^2+^, and Cu^2+^) was maintained at 0.33 mol/L^−1^. Simultaneously, a water immersion method was employed to test the pH values, and evaluate the impact of geopolymer cementitious materials on the environment. After curing for 28 d, the geopolymer cementitious materials were immersed in deionized water. The pH values of the immersion liquid were tested after 48 h, and the water was replaced. The pH values were then tested again after immersing for an additional 48 h. This procedure was repeated 10 times to observe the pH value variations.

## 3. Results and Discussion

### 3.1. Flowability and Setting Time

**Figure 4:**
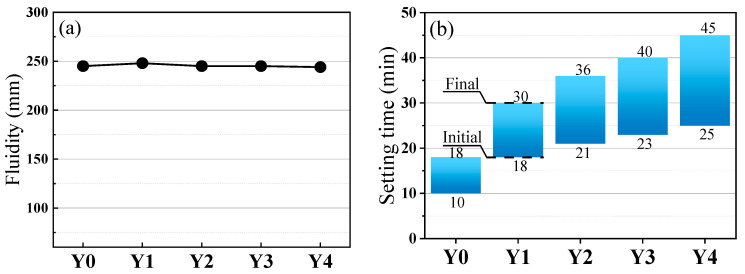
The workability of geopolymer cementitious materials: (**a**) fluidity; (**b**) setting time.

The flowability and setting time of geopolymer cementitious material slurry are illustrated in Figure 4. From Figure 4a, it can be observed that, under varying water-to-cement ratios, the flowability of the geopolymer cementitious material slurry remains relatively constant (244–248 mm) with the increase of YRS. The variation rate is less than 2%. The small change in mobility can effectively prevent variation in the thickness of the aggregate inclusion layer caused by excessive changes in the mobility of cementitious materials during the preparation of porous geopolymer concrete, which may affect the experimental data. From Figure 4b, it can be seen that the setting time of the geopolymer cementitious material slurry is gradually prolonged with the increase of YRS, and the initial and final setting time are 10 min and 18 min, respectively, when YRS is not added. When a small amount of YRS was incorporated (10%), the initial and final setting time of Y1 group is enhanced to 18 min and 30 min with enhancement rates of 80% and 66.7%, respectively. When YRS is 40%, the initial and final setting time of Y4 group is enhanced to 25 min and 45 min, with the enhancement rate as high as 150%. This demonstrates that the increase of YRS can significantly delay the setting time of the cementitious materials. This is due to the fact that the incorporation of YRS decreases the content of highly active RM and GGBS, which reduces the overall activity of the materials and hinders the generation of gel products. Therefore, the addition of YRS can improve the workability of geopolymer cementitious materials.

### 3.2. Compressive Strength

#### 3.2.1. Compressive Strength of Geopolymer Cementitious Materials

**Figure 5:**
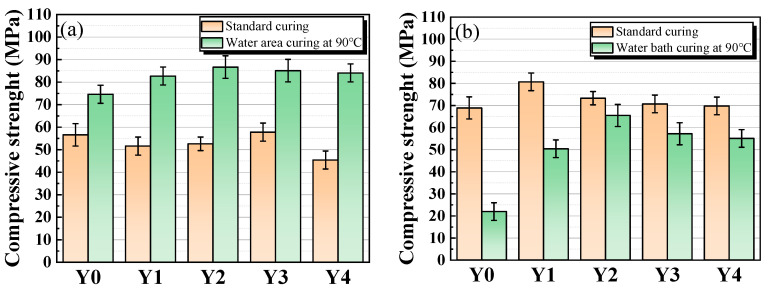
Compressive strength of geopolymer cementitious material under different curing conditions: (**a**) 3 d; (**b**) 28 d.

The compressive strength of geopolymer cementitious materials at an age of 3 d is shown in Figure 5a. Under standard curing, the compressive strength of geopolymer cementitious materials, except for the benchmark group, shows a tendency of increasing and then decreasing with the increase of YRS. In the benchmark group, both GGBS and RM have high activity, resulting in high compressive strength of the geopolymer cementitious materials. Due to the low activity of YRS in the natural state, when a small amount of YRS is added, the overall activity of raw materials in the ternary polymer decreases, resulting in a decrease in the compressive strength of geopolymer cementitious materials. With the increase of YRS, the content of Si in the C-A-S-H gel formed in the cementitious materials gradually increases, and the connection between the Si-O bond and the gel is gradually densified, which makes the structure of the C-A-S-H gel gradually stabilized, and the compressive strength of cementitious materials increases, reaching a peak value of 57.8 MPa at 30% of YRS dosages. When the dosage of YRS exceeds 30%, the stable state of C-A-S-H gel in the cementitious materials is destroyed, and the independent presence of excess SiO_2_ in YRS destroys the dense state of the gel and ultimately leads to the reduction of compressive strength. In the samples cured under high temperature water bath of 90 °C, the compressive strength of cementitious materials shows a tendency of increasing and then decreasing with the increase of YRS, and the highest compressive strength at an age of 3 d can reach 86.7 MPa. When a small amount of YRS is incorporated, the compressive strength of cementitious materials does not decrease, which is mainly due to the high-temperature and high-humidity environment prompting the depolymerization of SiO_2_ in YRS, which produces more reactive Si than that in standard curing to participate in the synthesis of the C-A-S-H products, thus improving the compressive strength. The peak compressive strength is achieved when YRS content reaches 20%, and the Si content in the C-A-S-H gel is moderate and the gel structure is stable. When YRS content exceeds 20%, the stability of the gel is gradually disrupted, leading to a gradual decrease in compressive strength. However, due to the increased efficiency of gel transformation at high temperature, a significant decrease in compressive strength is not observed even when YRS content exceeds 40%.

Figure 5b presents the compressive strength of geopolymer cementitious materials at an age of 28 d. The compressive strength under standard curing condition increases compared with those at an age of 3 d, but the peak compressive strength shifts from group Y3 to group Y1. Except for group Y0, the compressive strength of cementitious materials under standard curing decreases gradually with the increase of YRS. This is mainly due to the fact that the activity of YRS is lower than that of RM and GGBS, and the increase in YRS affects the conversion efficiency of the gel substance in cementitious materials, thus affecting the compressive strength. Compared with the group Y1~Y4, the group Y0 has only two types of raw materials for cementitious materials, and the particle size distribution is more dispersed, so it is difficult to form a more compact material particle stacking effect, which affects the compressive strength. From the compressive strength under high temperature water bath curing at age of 28 d in Figure 5b, with the increase of YRS, the compressive strength of cementitious materials shows the trend of increasing and then decreasing, which is consistent with the trend of the compressive strength change at an age of 3 d, but compared with the compressive strength at age of 3 d, the compressive strength of cementitious materials is reduced. This decrease in compressive strength is most pronounced in Y0 group. This is mainly due to the fact that the samples were kept at a high temperature of 90 °C during the first 3 d of curing, and the elements in the material, such as Ca, Al, and Si, rapidly reacted with water to form a high-density C-A-S-H gel. After 3 d, the sample is changed to standard curing of 20 °C, the surface of the specimen shrinks and internal temperature stress is generated. When the expansion stress is greater than the tensile stress, the specimen produces microcracks, thus affecting the compressive strength. This fragmentation is more obvious especially in Y0 group which has more gel production.

**Figure 6:**
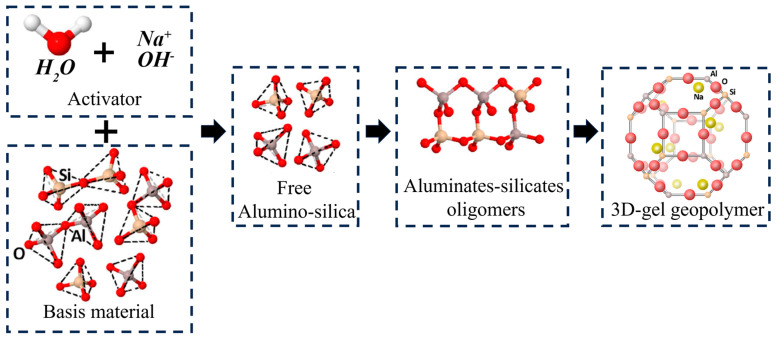
Strength formation mechanism of geopolymer cementitious materials.

The strength of geopolymer cementitious materials is mainly derived from the C-A-S-H gel, whose internal reaction mechanism is shown in Figure 6. When raw materials (RM, GGBS and YRS) containing elements, such as Si and Al meet OH^-^ in alkaline excitation solution, H^+^ depolymerizes and separates the Si and Al-containing oxides into free silica-oxygen tetrahedra and aluminum-oxygen tetrahedra, which are then reassembled to form the three-dimensional structure of C-A-S-H gels [41]. In comparison to the C-S-H gel in the traditional silicate system, this dense three-dimensional C-A-S-H gel structure exhibits superior mechanical properties [42].

#### 3.2.2. Compressive Strength of Porous Geopolymer Concrete

**Figure 7:**
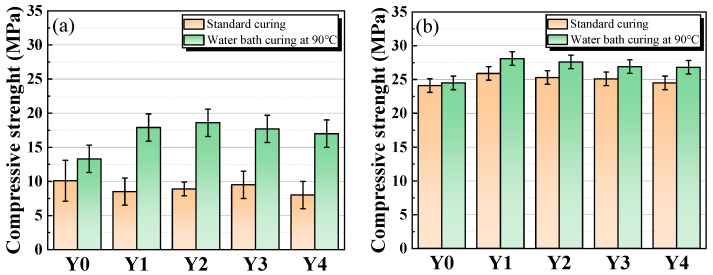
Compressive strength of porous geopolymer concrete under different curing conditions: (**a**) 3 d; (**b**) 28 d.

Figure 7 presents the compressive strength of porous geopolymer concrete at ages of 3 d and 28 d. From the compressive strength of porous geopolymer concrete at age of 3 d in Figure 7a, it can be seen that the compressive strength of cementitious materials tends to increase and then decrease with the increase of YRS under standard curing, except for the benchmark group. In high temperature water bath curing group, the compressive strength of cementitious materials shows a tendency to increase and then decrease with the increase of YRS. The increase in compressive strength of porous geopolymer concrete under the two curing conditions follows the same trend as that of cementitious materials, which is mainly due to the fact that the strength of porous geopolymer concrete is mainly derived from geopolymer cementitious materials used to bind the aggregates. From the compressive strength of porous geopolymer concrete at age of 28 d in Figure 7b, it can be seen that the compressive strength of porous geopolymer concrete decreases gradually with the increase of YRS, except for the benchmark group. The compressive strength of the high temperature water bath curing group is slightly higher than that of the standard curing group, which is mainly due to the fact that the high temperature and high humidity environment is more favorable for gel formation. The increase in temperature can convert some lower energy molecules into activated molecules and increase the number of effective collisions between molecules [28]. Meanwhile, the water bath environment provides a constant supply of OH^−^ for this reaction process [43]. These two factors accelerate the rate of reaction and increase the yield of gel, thus increasing the compressive strength of the samples. Overall, the 28 d compressive strength of porous geopolymer concrete does not vary much with different factors, ranging from 24.1 to 28.1 MPa. The overall trend in compressive strength of porous geopolymer concrete is essentially the same as that of geopolymer cementitious materials, which is mainly due to the fact that the reason for the trend in compressive strength is the same as that of geopolymer cementitious materials.

### 3.3. Porosity and Permeability Coefficient

**Figure 8:**
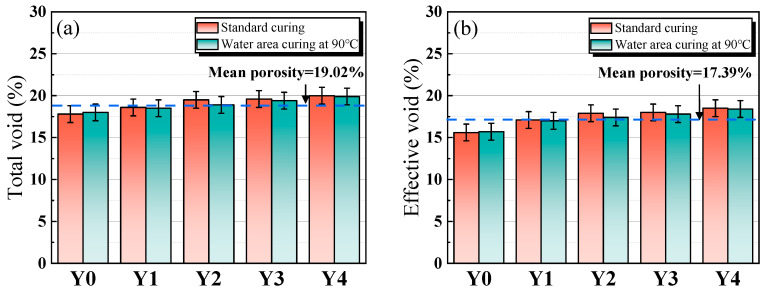
Total porosity (**a**) and effective porosity (**b**) of porous geopolymer concrete.

**Figure 9:**
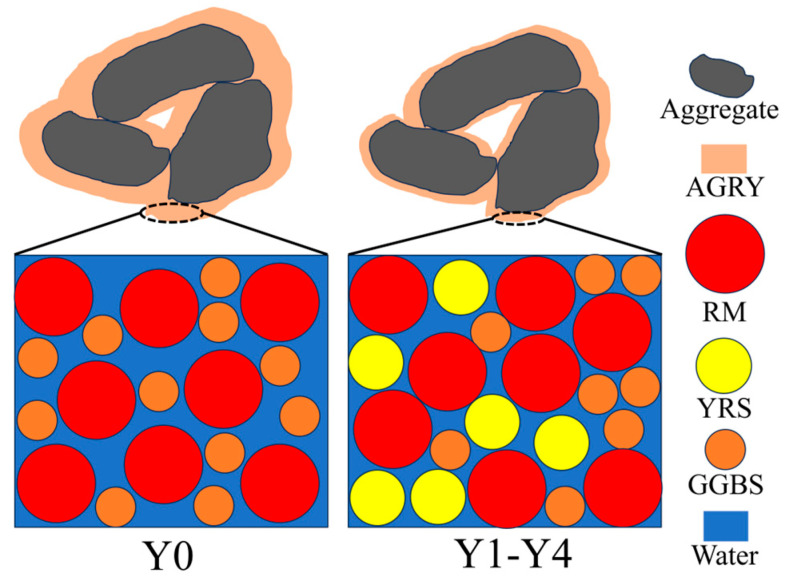
Particle accumulation of geopolymer cementitious materials.

Figure 8a,b show the total porosity and effective porosity of porous geopolymer concrete concrete, respectively. The porosity of porous geopolymer concrete increases slightly with the increase of YRS, and the total porosity changes from 17.8% to 20%, and the effective porosity changes from 15.6% to 18.5%. The average total porosity and average effective porosity of porous geopolymer concrete are 19.02% and 17.34%, respectively, which meets the criteria for the preparation of porous concrete with optimal porosity [35]. The total porosity measured for each sample is higher than the target porosity of 15%, which is mainly due to the increase in porosity caused by the roughness of the aggregate surface and the compaction process [44]. The increase in YRS leads to a gradual increase in porosity, which is mainly due to the distribution of particle sizes in matrix, and this reason can be explained by Figure 9. As shown in Figure 9, the particle size of YRS is located between RM and GGBS (shown Figure 1 in Section 2.1), which results in that when YRS is added to the Y0 benchmark group, the material has a more homogeneous particle size distribution and a denser micro-packing structure. This leads to a decrease in the overall solid volume of the aggregate encapsulating cementitious materials and ultimately leads to an increase in the porosity of the porous concrete.

**Figure 10:**
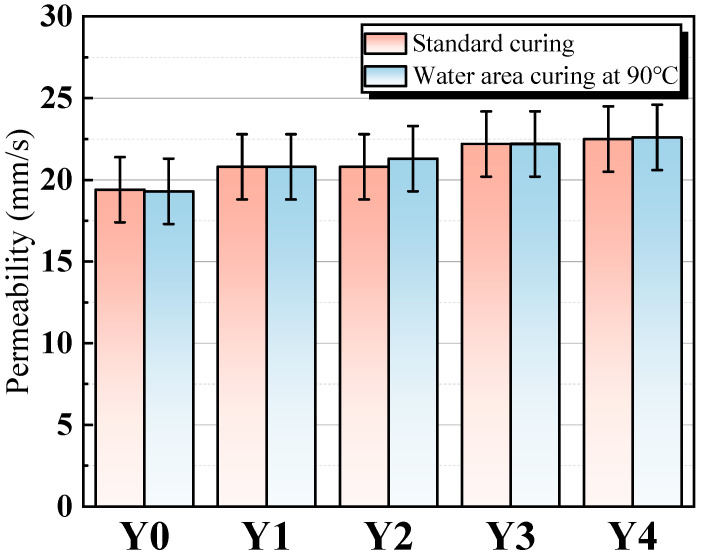
Permeability of porous geopolymer concrete.

Figure 10 shows the permeability of porous geopolymer concrete with various dosages of YRS. The permeability of porous geopolymer concrete tends to increase with the increase of YRS, but the increase is not significant. Comparing the water permeability of porous geopolymer concrete under the two curing conditions, there is no significant difference. The trend of gradual increase in permeability is similar to the trend of the increase in concrete porosity, and the reason for this is similar to the reason for the increase in porosity, which is caused by the increase in porosity due to the accumulation of aggregates encapsulated with cementitious materials. In conclusion, YRS and high temperature water bath curing have little effect on the permeability of porous geopolymer concrete, and the permeability rate remains between 19 and 22 mm/s.

### 3.4. XRD

**Figure 11:**
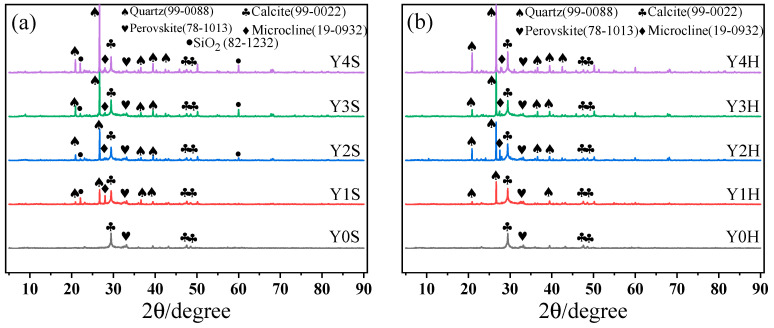
XRD patterns of geopolymer cementitious materials at age of 28 d: (**a**) standard curing; (**b**) water bath curing of 90 °C.

The XRD patterns of geopolymer cementitious materials at an age of 28 d under standard and high temperature water bath curing conditions are shown in Figure 11. First, the peaks of quartz, calcite, microplagioclase feldspar, chalcocite and silicon oxide can be observed. Then, it can be seen that the peaks of more obvious quartz appear near 27° in both the standard curing and high temperature water bath curing groups, and the peaks gradually increase with the increase of YRS. The quartz here is mainly derived from SiO_2_ in YRS. In addition, SiO_2_ in non-quartz form is observed near both 23° and 60° in the standard curing group, and these diffraction peaks increase with the increase of YRS. This is mainly due to the depolymerization of quartz during geopolymer formation allowing the transformation of silica-oxygen tetrahedra into SiO_2_. However, SiO_2_ was not found at this location in the high temperature water bath curing group, which is mainly due to the high temperature and high humidity environment that increases the reaction rate of the geopolymer, leading to the condensation reaction of SiO_2_ from quartz depolymerization, and ultimately incorporating into the C-A-S-H gel formed in geopolymer in the form of silica-oxygen tetrahedra. This demonstrates that SiO_2_ in YRS is involved in the geopolymer reaction. The elevation of the calcite peak near 30° in Figure 11 is related to the decomposition of calcium silicate in RM. The elevation of the calcite peak is also good evidence for the formation of C-A-S-H gel in the material. In the process of gel generation from this polymer, the main chemical reaction is the reaction of Ca^2+^, Si^4+^, and Mg^2+^ in the matrix with OH^-^ in the alkali activator as shown in Equations (6)–(8).

Ca^2+^ + CO_2_ + 2OH^−^ = CaCO_3_ (6)

Ca^2 +^ +2OH^−^ = CaO + H_2_O (7)

2CaO + Mg^2+^ +Si^4+^ = 2CaO•MgO•2SiO_2_ (8)

### 3.5. FT-IR

**Figure 12:**
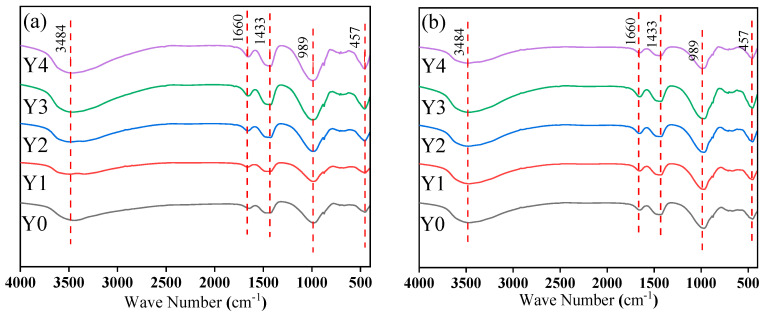
FT-IR spectrums of geopolymer cementitious material: (**a**) standard curing; (**b**) water bath curing of 90 °C.

Figure 12 shows the FT-IR spectrums of geopolymer cementitious materials with various doages of YRS and under different curing conditions. It can be seen that a significant characteristic peak, located at 457 cm^−1^, is revealed in all samples, which originates from the bending vibration of the Si-O bond in [SiO_4_]^4−^ [29]. In this band, the geopolymer cementitious materials show a shift towards lower wavelengths compared to raw materials, which is due to changes in crystallinity [45]. Meanwhile, a characteristic peak located at 989 cm^−1^, which corresponds to the asymmetric stretching vibrations of Si-O-Si and Si-O-Al, is observed in all samples. This indicates the formation of polymers in cementitious materials, which may be associated with the C-A-S-H structure [46]. Furthermore, a stretching band is observed near 1433 cm^−1^, which originates from the stretching vibration of C=O in the carbonate group and may be a characteristic band of calcite. In addition, a distinct characteristic peak at 1660 cm^−1^ is also observed, which is mainly caused by O-H vibration originating from water molecules adsorbed on the gel surface or carried in the raw material. All samples of geopolymer cementitious materials have the same peak position, indicating that the same chemical groups are produced during the reaction within geopolymer cementitious materials.

### 3.6. SEM

**Figure 13:**
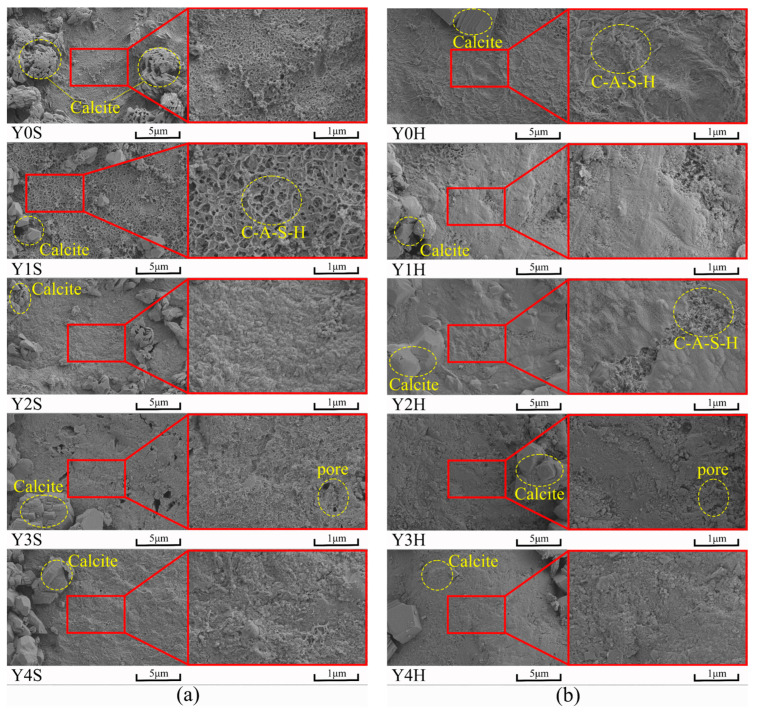
SEM images of geopolymer cementitious materials at age of 28 d: (**a**) standard curing; (**b**) water bath curing of 90 °C.

Figure 13 shows the morphology of geopolymer cementitious materials at an age of 28 d. It can be seen that under standard and high temperature water bath curing conditions, the geopolymer cementitious materials show similar morphology, and the surface of geopolymer cementitious materials is attached to a large area of gel products. From the left side of Figure 12a,b, it can be seen that large crystals are present in both cementitious materials, which are predominantly calcite. This is mainly due to the carbonation of calcium ions, as shown in Equation (6) in Section 3.4, which produces calcium carbonate when exposed to water. Meanwhile, with the increase of YRS, the microstructure of cementitious materials is gradually loosened and the unreacted YRS particles on the surface are gradually revealed. This is mainly due to the low activity of YRS, which is not fully reacted in cementitious materials. Furthermore, the holes in Y3S and Y3H groups are mainly caused by less gel production and results in a looser internal structure. The decrease in the internal structural compactness of cementitious materials caused by pores will seriously affect the compressive strength, which is consistent with the change rule of the compressive strength of cementitious materials in Figure 5.

### 3.7. Environmental Impact Assessment

#### 3.7.1. Heavy Metal Adsorption Capacity

**Figure 14:**
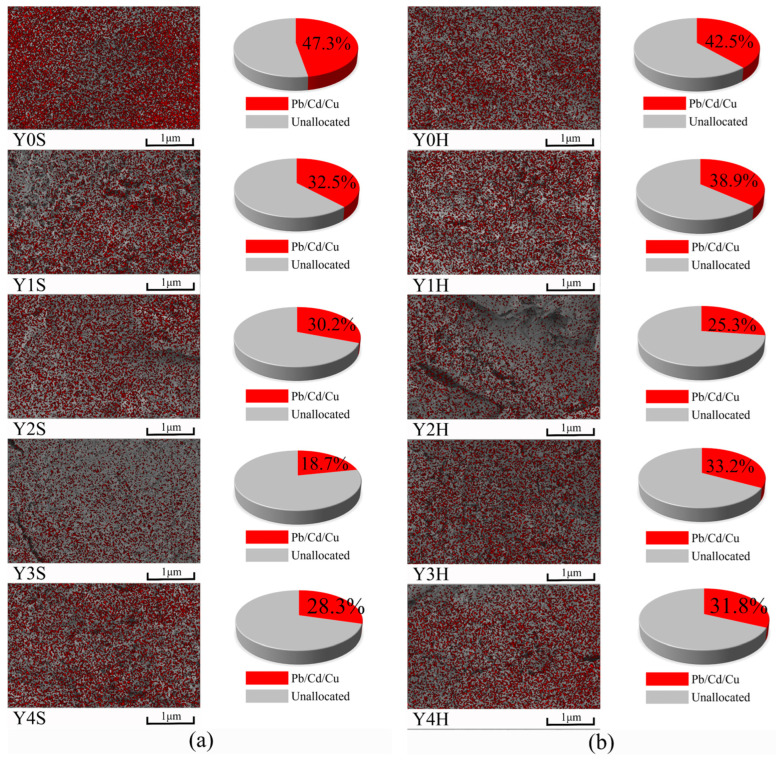
EDS of geopolymer cementitious materials adsorbed by heavy metals: (**a**) standard curing; (**b**) water bath curing of 90 °C.

Figure 14 shows the EDS scanning images of heavy metal adsorption on the surface of geopolymer cementitious materials. It can be seen that within the spectral scanning range, the gray area represents the unadsorbed portion and the red area represents the adsorbed heavy metals (including Pb, Cd and Cu), so the ratio of the area occupied by the red portion can be used to determine the adsorption effect. The image is datamined using Image J software version 1.54 and the heavy metal portion is selected using threshold segmentation, and then the area ratio is calculated. As can be seen in Figure 14, the heavy metal adsorption effect of geopolymer cementitious materials under both curing conditions shows a decreasing and then increasing trend with the increase of YRS. The areas occupied by the red areas in Figure 14a are 47.3%, 32.5%, 30.2%, 18.7% and 28.3%, and the areas occupied by the red areas in Figure 14b are 42.5%, 38.9%, 25.3%, 33.2% and 31.8%. This result is contrary to the results of compressive strength, where samples with higher compressive strength have weaker heavy metal adsorption capacity. This is mainly due to the fact that the cementitious material containing 40% YRS with lower compressive strength have less gel on the surface, which cannot fill the surface and have a higher specific surface area. This higher specific surface area makes it easier for heavy metals to remain on the surface of cementitious materials. In addition, the heavy metal adsorption amounts of Y0 group are the highest values, and the gel products of Y0 are also the most numerous, i.e., the heavy metal adsorption ions are mainly concentrated on the surface of C-A-S-H gel. From the above results, it can be seen that geopolymer cementitious materials possess good heavy metal adsorption effect.

#### 3.7.2. pH Values

**Figure 15:**
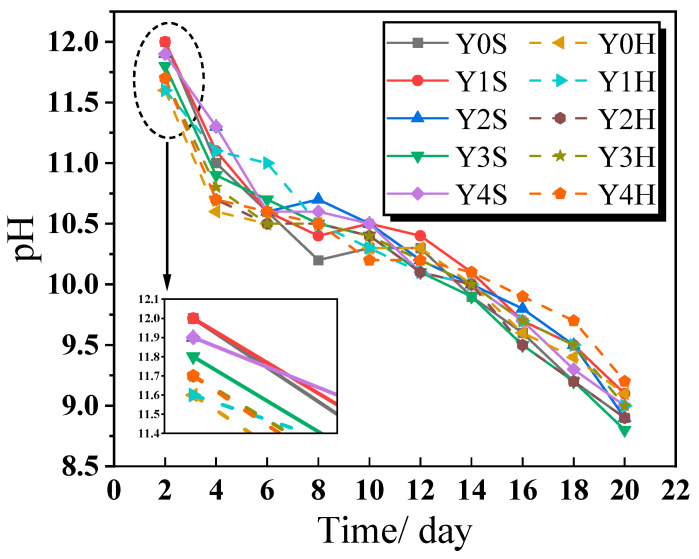
The pH values of the soaking solution after immersing the geopolymer cementitious material for different times.

Figure 15 shows the trend of the pH values of the soaking solution after immersing geopolymer cementitious materials for different times. As the soaking time increases, the pH values of the soaking solution gradually decreases. This is mainly caused by the gradual dilution of the alkaline substances in cementitious materials by neutral water. By observing the decreasing trend of pH, it can be seen that the decreasing rate can be divided into three parts, the decreasing rate of pH is faster at 0–6 d, the decreasing rate gradually slows down at 6–12 d, and the decreasing rate increases again after 12 d. The fast rate of pH decrease in the initial stage is mainly caused by the rapid scouring off of unreacted Na_2_SiO_3_ from the surface of cementitious materials by water immersion. Subsequently, during the gradual decline phase, the alkaline components in the unencapsulated RM particles on the surface of cementitious materials are slowly leached out. In the final stage, most of RM particles are removed from the surface of cementitious materials, and the protection of the internal gel inhibited further leaching of RM, thus lowering the pH values of the soaking solution. By observing the pH values of the first test (shown in Figure 15 for a partial enlargement), it can be seen that the alkalinity of the soaking solution of cementitious materials under standard curing is higher than the alkalinity of the soaking solution of cementitious materials under high temperature water bath curing. This is mainly due to the fact that the alkali excitation reaction of cementitious materials under high temperature water bath curing is more complete, and the alkali exciters on its surface are diluted into the water, so that the hot water containing alkali accelerates the hardening on the surface of cementitious materials. This will inevitably consume the alkali stimulant on the surface of cementitious materials, thus lowering the pH values in the early stage. This experiment simulates the environmental impacts of pavement in a rainfall environment. As the number of rainfall days increases, the pH of the rainwater soaked through the pavement gradually returns to neutral, and its impact on the environment gradually decreases.

## 4. Conclusions

The aim of this work is to prepare a geopolymer cementitious material with good working performance and high compressive strength, and on this basis, to prepare a porous geopolymer concrete with environmentally friendly properties. Through the macroscopic performance tests and microscopic mechanism analysis, the main conclusions are as follows:

(1) The addition of YRS can improve the working performance of geopolymer cementitious materials, and the increase of YRS can significantly delay the setting time of geopolymer cementitious materials. When the dosage of YRS is 40%, the initial and final setting time of geopolymer cementitious materials is increased to 25 min and 45 min, respectively, which is up to 150% improved compared with that of no YRS. The fluidity of the prepared geopolymer cementitious material slurry is maintained at 244–248 mm with a variation of less than 2%.
(2) The compressive strength of both geopolymer cementitious materials and porous geopolymer concrete tends to increase and then decrease with the increase of YRS. The water bath curing of 90 °C can accelerate the early compressive strength of geopolymer cementitious materials, and the highest compressive strength up to 86.7 MPa at an age of 3 d. The porous geopolymer concrete obtains a compressive strength of up to 28.1 MPa at an age of 28 d.
(3) The water bath curing of 90 °C has less of an effect on the porosity and permeability coefficient of porous geopolymer concrete. With the increase of YRS, the total porosity and effective porosity of porous geopolymer concrete show a gradual increase, and its average total porosity and average effective porosity are 19.02% and 17.34%, respectively. The total porosity of the prepared porous geopolymer concrete is higher than the target porosity of 15%, which meets the requirement of optimal water permeability.
(4) The gel products in geopolymer cementitious materials are mainly C-A-S-H gels, and YRS participates in the geopolymer reaction. The high temperature water bath curing of 90 °C promotes the participation of YRS in the geopolymer reaction to generate C-A-S-H gels, which produces a large amount of calcite. The water bath curing of 90 °C and the addition of YRS do not affect the alteration of the chemical groups of geopolymer cementitious materials.
(5) The geopolymer cementitious materials have good heavy metal adsorption, and the heavy metal adsorption rate is related to the amount of gel products and the morphology of cementitious materials. The alkaline substances in geopolymer cementitious materials can be diluted by prolonged soaking in water, and their damage to the environment can be mitigated.

## Figures and Tables

**Table 1:** Chemical compositions of RM, GGBS and YRS (wt%).

Oxide (wt%)	CaO	SiO_2_	Fe_2_O_3_	Al_2_O_3_	TiO_2_	Na_2_O	SO_3_	K_2_O	MgO	LOI
RM	48.76	22.05	9.46	7.90	4.91	3.81	0.81	0.33	1.31	0.66
GGBS	49.39	25.41	0.29	14.16	2.13	0.32	2.02	0.43	5.41	0.34
YRS	8.90	65.67	4.76	10.87	0.85	2.79	0.10	2.77	2.62	0.67

**Table 2:** The mixture ratio of materials required per liter of porous geopolymer concrete (kg/m^3^).

Specimen	GGBS/kg	RM/kg	YRS/kg	Alkali/kg	Water/kg	Aggregate/kg	w/c
Y0-S Y0-H	224	96	/	54.4	128	1600	0.4
Y1-S Y1-H	201.6	86.4	32	54.4	121.6	1600	0.39
Y2-S Y2-H	179.2	76.8	64	54.4	115.2	1600	0.38
Y3-S Y3-H	156.8	67.2	96	54.4	108.8	1600	0.37
Y4-S Y4-H	134.4	57.6	1128	54.4	102.4	1600	0.36

Note: S is standard curing and H is high temperature water bath curing.

## Data Availability

Data are contained within the article.
